# Therapeutic Monitoring of Vancomycin Implemented by Eremomycin ELISA

**DOI:** 10.3390/antibiotics13121133

**Published:** 2024-11-25

**Authors:** Inna A. Galvidis, Yury A. Surovoy, Vitaly R. Sharipov, Pavel D. Sobolev, Maksim A. Burkin

**Affiliations:** 1 I. Mechnikov Research Institute for Vaccines and Sera, Moscow 105064, Russia; galvidis@yandex.ru; 2 University College of London Hospital, London NW1 2BU, UK; ysurovoy@gmail.com; 3 Exacte Labs LLC, Moscow 117246, Russia

**Keywords:** vancomycin, eremomycin, immunoassay, pharmacokinetics, therapeutic drug monitoring

## Abstract

Background/Objectives: Due to a narrow therapeutic window, side-effects, toxicities, and individual pharmacokinetics (PK) variability, WHO classifies vancomycin (VCM) as a “watch antibiotic” whose use should be monitored to improve clinical effectiveness. Availability and ease of use have made the immunoassay technique the basic tool for the therapeutic drug monitoring (TDM) of VCM concentrations. Methods: The present study describes the development of a TDM tool for VCM based on anti-eremomycin (ERM) antibody enzyme-linked immunosorbent assay (ELISA). Results: The optimized assay format based on coating a BSA-VCM conjugate allowed for the equal recognition of both VCM and ERM (100 and 104%) and was not influenced by concomitant antibiotics. Among the sample pretreatments studied, acetonitrile deproteinization was preferred to effectively remove the most likely matrix interferences and to provide 75–96% VCM recovery in the range of 3–30 mg/L, ensuring reliable determination of the key PK parameter, Ctrough. Higher peak concentrations were measured in more diluted samples. Several inflammatory indices, biochemical markers, and key proteins significantly different from normal in critically ill patients were investigated as assay interferers and were found not to interfere with VCM analysis. Serum samples (n = 108) from patients (n = 4) with extensive burn injuries treated with combined antibiotic therapy were analyzed for VCM using the developed assay and confirmed by LC-MS/MS, demonstrating good agreement. Conclusions: The approach used shows that the same analytical instrument is suitable for measuring structurally related analytes and is fully adequate for their therapeutic monitoring. Suboptimal exposure based on Ctrough values obtained with standard dosing regimens supports the use of TDM in these patients.

## 1. Introduction

Vancomycin (VCM) is a relatively old glycopeptide antibiotic, which still has a major role in the treatment of pneumonia, septicemia, and skin and tissue soft infections, as well as infectious endocarditis caused by methicillin-resistant Staphylococcus aureus (MRSA). Despite several newer and less toxic drugs on the market, including other glycopeptides, oxazolidinones, lipopeptides, and glycylcyclines, VCM remains a first-line therapy for many of these indications (especially angiogenic and catheter-related infections) in the guidelines issued by the infectious disease society of America [1], the British Society for Antimicrobial Chemotherapy [2], and the Strategy of Antimicrobial Therapy Control in Russia [3]. Vancomycin is characterized by poor absorption from the gastrointestinal tract and is mainly used intravenously. Available efficacy and toxicity data suggest that optimal vancomycin exposure based on the area under the curve over 24 h (AUC24h) lies between 400 and 600 mg·h/L [1,4]. Minimal VCM concentration (C_trough_) is sometimes used as a surrogate of AUC with values below 10 mg/L associated with reduced VCM exposure [5]. VCM demonstrates significant pharmacokinetic variability, for example, obesity and increased total body weight are accompanied by a higher VCM volume of distribution [6], and augmented renal clearance commonly encountered in patients with sepsis, burn injuries, and hematological malignancies [7,8] promotes increased VCM elimination, leading to a significant risk of VCM under-exposure [9]. Decreased renal function, aging [10], and hypoalbuminemia [11], on the contrary, might predispose patients to high VCM concentrations and toxic effects. Given the complexity of all these factors, determining VCM pharmacokinetics in critically ill patients through therapeutic drug monitoring is recommended to improve safety and efficacy [12].

A variety of analytical techniques are employed to quantify vancomycin in plasma or serum. Along with simple, inexpensive, but poorly specific microbiological methods based on the growth inhibition of VCM-susceptible *Bacillus subtilis* test-strains [13] and specific, accurate, but complex, expensive, and less accessible chromatographic methods [14], immunoassays have become the most widely demanded tools for the therapeutic drug monitoring (TDM) of VCM since its clinical introduction.

Radioimmunoassay (RIA) [15], fluorescence polarization immunoassay (FPIA) [16], enzyme multiplied immunoassay technique (EMIT) [17], particle-enhanced turbidimetric inhibition assay (PETINA) [18], competitive chemiluminescent immunoassay (CMIA), and kinetic interaction of microparticles in a solution (KIMS) [19] are the known types of immunoassays for the TDM of VCM, which differ in the marker molecule (radioisotope, fluorophore, enzyme, or microparticles) and the type of output signal. To monitor the concentration of vancomycin in serum, a lateral flow immunoassay format based on chelated europium (III) nanoparticles [20] and an electrochemical aptamer-based sensor [21] have also been proposed. Such aspects as high sensitivity and specificity, high throughput, relatively low cost, and simple and fast handling make immunoassays attractive for TDM. However, the advantage of a fast measurement is achieved by homogeneous interactions and analysis of the initial sample without any separation, pretreatment, or dilution in most of the assay types mentioned. This makes them more susceptible to sample matrix interference, which can cause false results [22,23,24]. Such negative matrix interference is thought to result from paraproteins, multispecific immunoglobulins, C-reactive protein, rheumatoid factor, or other unknown endogenous substances [25,26]. Although another type of assay, the enzyme-linked immunosorbent assay (ELISA), is more time-consuming (1.5–2.5 h) than the previous ones because it is performed in several steps, separating the interactions limits the influence of the matrix.

**Figure 1:**
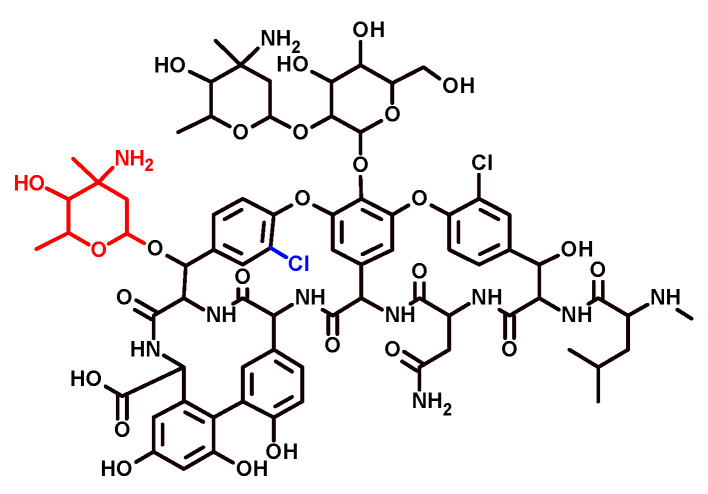
Chemical structure of VCM and ERM glycopeptides. The black formula is the general backbone. The 4-epi-vancosamine (red) is the characteristic substituent in ERM. Additional chlorine (blue) is present in VCM.

In the present study, based on a previously established specificity-tuning approach [27,28,29], we developed a TDM tool for VCM using an antibody against its structural analog eremomycin (ERM) (Figure 1). Antibodies against common ERM/VCM epitopes were selected by binding to VCM-modified coating antigens, thus providing VCM recognition. The influence of potential factors in the serum samples from critically ill patients, which are suspected to interfere with VCM immunoassays and lead to erroneous results, was given special attention in the current work.

## 2. Results and Discussion

### 2.1. ELISA Performance for VCM Quantification

**Figure 2:**
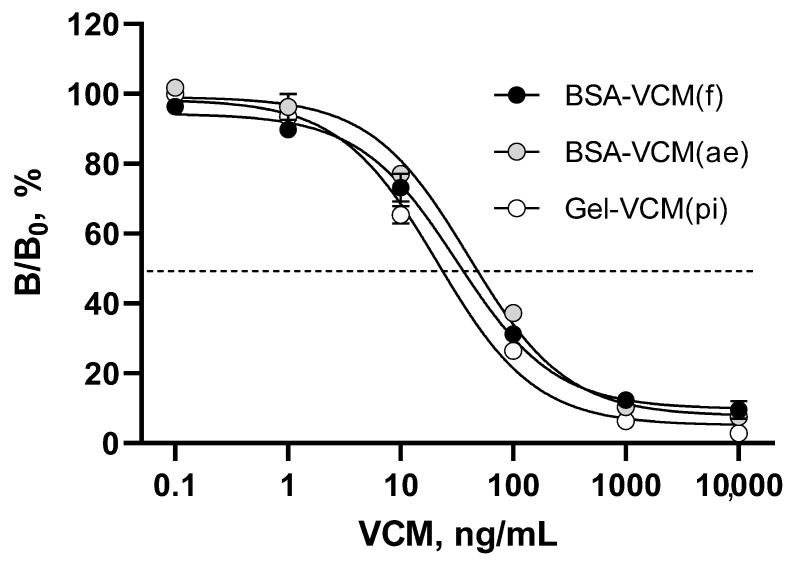
Standard curves and analytical parameters of VCM determination in ELISAs based on coating VCM conjugates. Each point is represented by the average value (n = 3) ± standard deviation.

Based on our previous studies on the hapten modification approach [27], it can be assumed that coated VCM structurally related to an immunizing hapten (ERM) is able to selectively interact with antibodies against common structural epitopes from the antiserum against ERM conjugated to glucose oxidase (GO-ERM) and thus switch the selective assay to recognize both analytes. Thus, heterologous VCM-based coating antigens made it possible to quantify VCM in ELISAs using anti-ERM antibodies as the recognition element. We examined three VCM conjugates for plate coating that had provided the highest cross-reactivity of VCM. The optimized antigen/antibody ratios found by the checkerboard titration method allowed the following calibrations to be generated (Figure 2) and the analytical parameters of the assays to be compared (Table 1).

The similarity of the analytical characteristics of the considered assay variants, which differed by only two-fold, was not a sufficient criterion to select the best among the suitable ones. Therefore, these three ELISAs were investigated and compared in recovery experiments.

### 2.2. Examination of VCM Recovery from Fortified Human Serum from Heath Volunteers

The accuracy of the assay was assessed in the range of concentrations of 3–30 mg/L, which was selected to include the recommended C_trough_ range of 15–20 mg/L [30]. Higher concentrations are associated with an increased risk of nephrotoxicity [31], while C_trough_ < 15 mg/L was shown to correspond to a higher risk of treatment failure [32]. To this end, several sample pretreatment approaches were attempted to estimate VCM recovery from healthy volunteer serum. The most commonly used protein precipitants such as methanol (MeOH), acetonitrile (ACN), and trichloroacetic acid (TCA) [33] were compared with assay buffer PBST (0.15 M phosphate-buffered saline containing 0.05% Tween 20, pH 7.2), used for serum dilution (Table 2).

Of note, VCM extraction from human serum with MeOH and TCA for serum deproteinization is quite low. The respective recoveries of 15–60% and 4–63% in all ELISA variants were considered inadequate, whereas ACN deproteinization resulted in a sufficiently better recovery rate of 49–96%. Serum dilution with PBST was also suitable and gave 89–117% recovery. Summarizing the data in the table, the most acceptable BSA-VCM×50f ELISA and sample pretreatments, ACN deproteinization and PSBT dilution, which provided adequate recoveries of 75–96% and 89–100%, with coefficients of variation not exceeding 9.7% and 11.7%, respectively, were reliable for VCM quantification and were selected for subsequent experiments.

The same samples spiked with VCM were frozen and analyzed by HPLC-MS/MS one week later. The recovered concentrations confirmed excellent accuracy of 99–101% with a coefficient of variation < 6% (Table S3) and stability of the analyte in human serum over this period over several freeze/thaw cycles.

### 2.3. Examination of Matrix Factors from Patient Sera on Assay Performance

**Figure 3:**
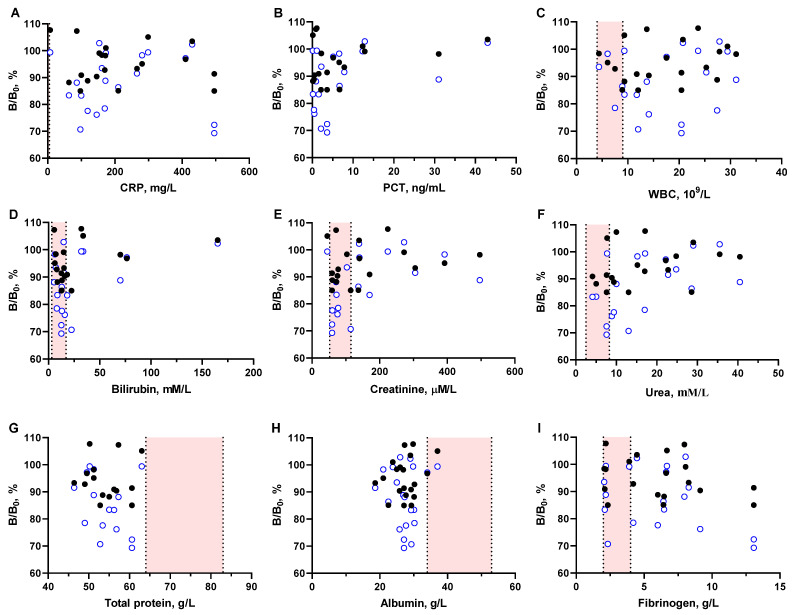
Effect of key clinical parameter values in serum samples from critically ill patients (n = 20) without VCM therapy on ELISA output signal (100%). Normal ranges for C-reactive protein (**A**), procalcitonin (**B**), white blood cells (**C**), bilirubin (**D**), creatinine (**E**), urea (**F**), total protein (**G**), albumin (**H**), and fibrinogen (**I**) are indicated with pink. Sera samples were deproteinized with ACN (filled circle) and diluted with PBST (empty circle). Each point is represented by the mean value (n = 3).

A number of studies report inaccurate results with commercial immunoassay systems for VCM [22,23,24,25,26]. Suspected interfering factors include C-reactive protein, immunoglobulins, or immune complexes such as rheumatoid factor. To account for this possible interference, we examined the matrix effect of sera from a non-VCM group of critically ill patients with varying levels of different proteins and other possible interfering factors and plotted them against the antibody binding levels (100%) (Figure 3).

**Figure 4:**
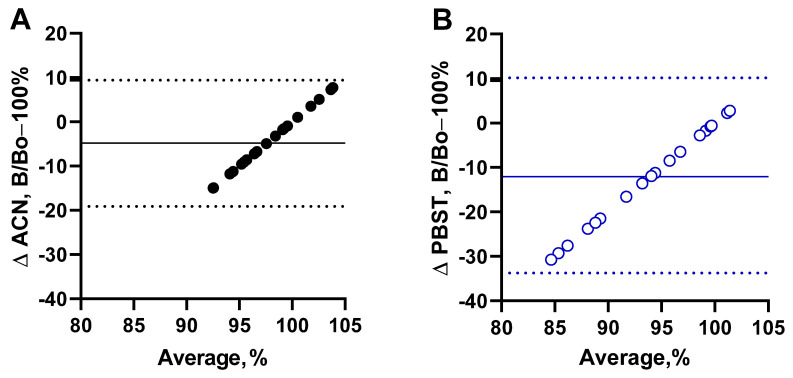
Comparative Bland–Altman plots for interference from sera of critically ill patients (n = 20) without VCM therapy on the output signal. Samples were pretreated by deproteinization with ACN (**A**) and dilution with PBST (**B**). Solid lines are biases of 100% antibody binding, while dotted lines are 1.96 × SD.

It is noteworthy that such indicators of inflammation as C-reactive protein (CRP), procalcitonin (PCT), and lymphoid cells (WBC) (Figure 3A–C), the biochemical markers bilirubin, creatinine, and urea (Figure 3D–F), as well as protein composition (Figure 3G–I) were significantly different from normal levels (pink range) and varied widely in critically ill patients. Furthermore, the graphs show that changes in these parameters do not correlate with antibody binding levels. Thus, there was no concentration-dependent effect of the considered factors on the output signal of the developed assay. However, compared to simple sample dilution (empty blue circles), there was an evident positive effect of serum deproteinization (black dots), which avoids possible protein-mediated interference. This is more clearly illustrated by the Bland–Altman plots (Figure 4). A comparison of the two pretreatment procedures showed that the ACN-depleted serum samples showed less bias compared to the control (Figure 4A). Due to the reduced matrix effect on antibody binding, such an approach should provide more accurate results.

### 2.4. Assay Specificity Examination

The anti-ERM antibody was utilized in the present study to quantify VCM. The special selection of heterologous coating antigen promoted the equal recognition of both, immunizing hapten ERM and target analyte VCM as 104 and 100%, respectively. A recent report found that related cross-reacting lipoglycopeptides could interfere with the accurate quantification of VCM in the enzyme-multiplied immunoassay technique (EMIT), particle-enhanced turbidimetric inhibition immunoassay (PETINIA) [34]. In the present study, the assay detects only VCM and ERM equally, but the clinical situation of the co-administration of several glycopeptides such as ERM, VCM, or other members of this antibiotic family is extremely unlikely. Due to antibody recognition of the common moiety of ERM/VCM molecules opposite the conjugation site (ERM amines) in the immunogen, the developed assay failed to recognize the related glycopeptide teicoplanin (<0.3%) with a substituent in this target epitope region. The same region of the molecule is also modified in the crystalline degradation product of VCM, CDP-1, which likely makes it non-cross-reactive in the present assay. This inactive product is known to be a factor in causing false-positive results in a number of VCM immunoassays due to cross-reaction with anti-VCM antibodies [35].

Other structural analogs, glycoside-containing and/or concomitant antibiotics, did not show cross-reactivity (Table 3). Therefore, they could not be recognized and had no effect on VCM quantification.

### 2.5. VCM Quantification Using ELISA and LC-MS/MS

**Figure 5:**
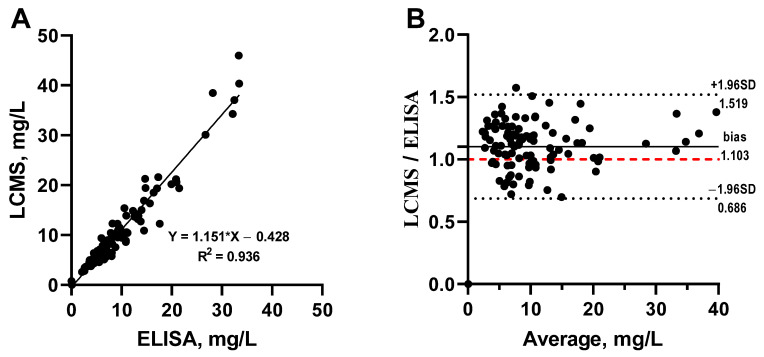
Two methods’ correlation (**A**) and Bland–Altman plot (**B**) of VCM concentrations in sera samples (n = 108) from 3 thermic trauma patients measured with ELISA and LC-MS/MS. The 95% limit of agreement interval is indicated with dotted lines: upper limit (mean + 1.96 × SD) and lower limit (mean *−* 1.96 × SD).

Serum samples from three patients (n = 108) were tested in the developed ELISA and LC-MS/MS to establish the agreement between methods. Both concentration groups were highly correlated (R^2^ = 0.936) (Figure 5A).

The Bland–Altman plot showed that ratios between the two methods’ measurements were within the 95% agreement interval, with a low mean bias of 0.103 indicating acceptable inter-assay agreement (Figure 5B). Accordingly, the correlation and confirmation of the data using the LC-MS/MS reference method demonstrated that the developed ELISA is a reliable method, providing good accuracy for the quantitative determination of VCM in human serum. Furthermore, its availability, low cost, and ease of operation without the need for specially trained personnel make it a more attractive option compared to LC-MS/MS.

### 2.6. VCM Pharmacokinetics in Patients with Major Burns

**Figure 6:**
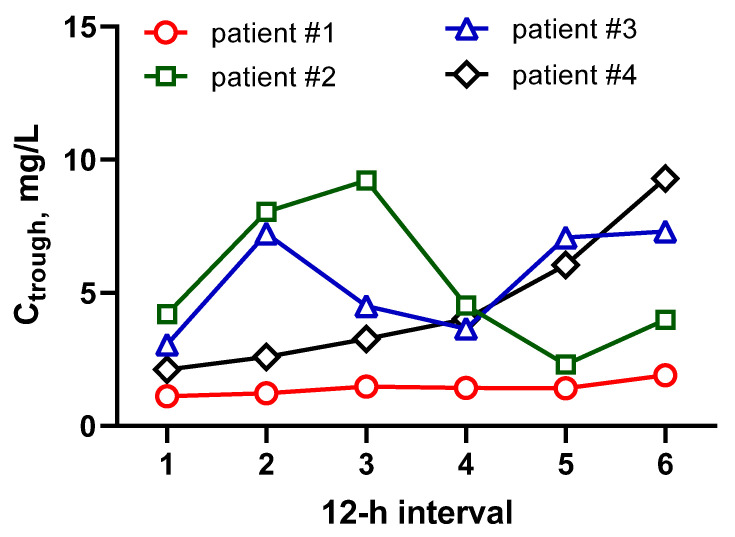
Approximation of Ctrough in four critically ill patients with major burns. Each point represents the lowest concentration within each 12 h dosing interval.

VCM serum concentrations were obtained in four critically ill patients with major burns and secondary infections for whom blood samples were collected previously within the separate TDM project for cefoperazone-sulbactam (Figure S3). As the sampling time points were not specifically targeted for VCM analysis, pharmacokinetic analysis was not performed. The lowest concentrations at each 12 h dosing interval were selected as the closest approximation of the Ctrough, and these concentrations are represented in Figure 6.

The median Ctrough approximated value in our study was 3.8 mg/L (90% CI (90% CI 3.1–4.6), which indicates a high risk of under-dosing in this cohort of patients. This keeps in line with the previous data on VCM which demonstrated increased clearance and suboptimal exposure in critically ill patients with burns. The suspected mechanisms of this phenomenon include augmented renal clearance and increased tubular secretion of VCM in patients with burns [36,37]. This is a plausible explanation for low VCM serum concentration in our patients, who were relatively young (29–46 years) and had high creatinine clearance (94–153 mL/min) (Table S4).

## 3. Materials and Methods

### 3.1. Chemicals and Materials

Vancomycin hydrochloride (VCM) was purchased from ICN Biomedicals (Aurora, OH, USA). The Gause Institute of New Antibiotics kindly provided eremomycin (ERM). Polyclonal antibodies against ERM were prepared in rabbits immunized with ERM conjugated to glucose oxidase using the glutaraldehyde method (GO-ERM) [38]. Bovine serum albumin (BSA), formaldehyde (f), 1-ethyl-3-(3-dimethylaminopropyl) carbodiimide (EDC), N-hydroxysuccinimide (NHS), sodium periodate (pi), and sodium borohydride were from Sigma (St. Louis, MO, USA). Dimethylformamide (DMF) was from Serva (Heidelberg, Germany). Gelatin (GEL) was a product of Bio-Rad (Hercules, CA, USA). Goat anti-rabbit IgG conjugated to horseradish peroxidase (GAR-HRP) was purchased from Imtek (Moscow, Russia). High-binding polystyrene 96-well plates were from Costar (Corning, Durham, NC, USA).

The coating antigens were adsorbed on the plates in 0.05 M carbonate–bicarbonate buffer (CBB, pH 9.6). The washing plates from unbound reagents and the dilution of standard and samples were conducted using 0.15 M phosphate-buffered saline containing 0.05% Tween 20 (PBST, pH 7.2). Antibody and GAR-HRP were prepared in 1% BSA-PBST. The 3,3′,5,5′-tetramethylbenzidine (TMB) substrate solution was provided by Bioservice (Moscow, Russia). To terminate the enzymatic reaction, 1 M H_2_SO_4_ was used.

### 3.2. Synthesis of Conjugated Antigens

The conjugation of VCM to protein carriers such as BSA and GEL was achieved by using three different coupling methods. The detailed procedures were described in [26]. Formaldehyde condensation (f, Mannich reaction) allowed the amine groups to couple with active hydrogen molecular sites [39]. The mixture of BSA (4 mg, 0.06 μmol) and VCM (4.35 mg, 3 μmol) was supplemented with 0.3 mL of 37% formaldehyde, stirred overnight at 37 °C, and then dialyzed exhaustively against PBS. Thus, BSA-VCM×50f was prepared with a protein/VCM coupling ratio of 1 to 50.

The carboxyl group of VCM was activated by NHS/EDC to conjugate with protein amines using the activated ester method (ae). VCM (4.35 mg, 3 μmol) was mixed with 4.5 μmol NHS and EDC to form 1 mL solution in DMF and stirred for 1.5 h at RT. The activated VCM was then added dropwise to BSA (4 mg, 0.06 μmol) in 1 mL CBB and conjugated for 3 h with stirring to give BSA-VCM×50ae.

The third method involved periodate (pi) oxidation of glycoprotein (GEL) conjugated to VCM amines to produce Gel-VCM×25pi. Crystalline NaIO_4_ (1.07 mg, 5 µmol) was dissolved in 1 mL of 10 mM acetate buffer (pH 5.0) containing 8 mg GEL (50 nmol) and stirred for 15 min with a magnetic stirrer. After overnight dialysis against 5 L of 10 mM acetate buffer (pH 5.0) at 4 °C, the resulting oxidized glycoprotein was added to VCM (1.8 mg, 1.25 µmol) in 1 mL CBB. The mixture of carrier and hapten, taken at a molar ratio of 1/25, was stirred for 2 h and then for another 2 h after the addition of NaBH_4_ (0.1 mL, 2 mg/mL). Excess reagent was removed by exhaustive dialysis against PBS.

The immunochemical activity of the prepared antigens coated on the plates confirmed successful conjugation.

### 3.3. Competitive Indirect ELISA

The assay procedure, calibration, recording, and calculation of results did not differ from our previous report on glycopeptides [40]. The coating conjugates were adsorbed in 96-well plates from solutions (0.05–1.5 µg/mL) in 0.1 mL CBB. After overnight incubation at 4 °C, the plates were washed three times with PBST to remove unbound reagent. Serial dilutions of antibodies in 0.1 mL 1% BSA-PBST were combined with standard (0, 0.01–10,000 ng/mL) in 0.1 mL PBST and incubated for 1 h at 25 °C. Following the washing procedure, the bound antibody was then detected using the GAR-HRP (1 h, 37 °C) in 0.1 mL of 1% BSA-PBS and subsequently washed out. The enzymatic activity was determined by the addition of TMB substrate (0.1 mL) to the wells, followed by the addition of 0.1 mL of 1 M sulfuric acid 30 min later, which terminated the reaction. The absorbance of the colored reaction product was read at 450 nm using a LisaScan spectrophotometer (Erba Mannheim, Karásek, Czech Republic).

The maximum optical output signal (100%) at zero concentration of VCM was referred to as B_0_. The decrease in the signal caused by each antibiotic concentration was expressed as the B/B_0_ ratio and was used to construct standard curves using a four-parameter logistic fitting in GraphPad Prism 8.0 software. The VCM half-inhibitory concentration (IC_50_) served as the sensitivity value for the comparison of assay variants and the cross-reactivity of analogs. The dynamic range of the assay was accepted as IC_20_-IC_80_, and the limit of detection reliably differing from B_0_ (≥3SD) was set as 10% of the inhibitory concentration value (IC_10_).

The checkerboard titration was employed to identify the optimal ratio of coating antigen to antibody, with concentrations taken in a serial fashion. A series of reagent combinations providing binding absorbances between 0.8 and 1.2 were evaluated in order to identify a more sensitive assay option based on a comparison of their IC_50_ values.

### 3.4. Sample Collection

Serum samples were collected from critically ill patients (n = 4) with major burns and secondary bacterial infections who received VCM as part of their empirical antimicrobial therapy. VCM was administered intravenously at a 1 g dose every 12 h in combination with cefoperazone/sulbactam according to the national recommendations for the empirical antimicrobial therapy of infections caused by multidrug-resistant microorganisms [41].

Blood samples were collected over the period of 72 h at time points not specifically matched to the expected VCM pharmacokinetic curve (blood samples were initially collected as part of the different drug TDM). Non-VCM serum samples were obtained from critically ill patients (n = 20) previously enrolled in our PK studies [42] along with the clinical data recorded for these patients. Informed consent forms were signed by the patients or their legal representatives. The study was approved by MEDSI Clinic Independent Ethical Committee, Moscow, Russia (Protocol #29 15 April 2021).

### 3.5. Sample Pretreatment and Recovery Examination

Frozen serum samples were thawed prior to analysis and assayed for VCM concentration by ELISA and LC-MS/MS in parallel. The procedure and sample preparation for LC-MS/MS analysis are detailed in the Supplementary File.

Blank sera samples from healthy volunteers were spiked with VCM at 3, 10, and 30 mg/L and incubated for 1 h at 37 °C. Before the analysis of VCM concentration, serum samples were thawed, diluted with PBST, or deproteinized using MeOH, ACN, or TCA. The precipitate was sedimented by centrifugation for 5 min at 6800× *g*, and the resulting supernatant appropriately diluted with PBST was tested by ELISA.

To estimate the preferred sample pretreatment procedure, assay accuracy, and precision, the recovery rate in spiked samples taken for analysis in four replicates was determined as the percentage ratio of the measured VCM concentration to the spiked VCM concentration.

## 4. Conclusions

VCM is a common and widely used antibiotic against Gram-positive infections. VCM has a narrow therapeutic window, a number of side effects and toxicities, and individual PK variability. Therefore, VCM is a drug whose exposure should be monitored by TDM for optimization. The present report is an attempt to develop an immunoassay TDM tool for VCM based on an antibody against a structural analog, eremomycin. Similar resource-saving approaches, which employ established or commercial assay systems to identify compounds with structural similarity through cross-reactions, have been documented in the literature [28,43,44]. In addition, some commercial systems based on homogeneous VCM immunoassays are more susceptible to potential interference and have been the subject of complaints, whereas the introduction of a heterogeneous immunoassay could likely negate this drawback. To address this issue, three competitive ELISA formats using heterologous coating conjugates of different designs were evaluated for optimal analytical parameters, recovery rate, and sample pretreatment procedure. The selected assay variant based on coating BSA-VCM×50f allowed equal recognition of VCM and ERM (100 and 104%, respectively) and has no cross-reaction from other structural analogs or concomitant antibiotics. Special attention was paid to the study of possible interference in the immunochemical interaction by some factors in the matrix of sera from critically ill patients and the search for ways to overcome them. Among the sample pretreatments studied, ACN-mediated deproteinization was preferred to effectively remove the most likely matrix interferences and to provide 75–96% VCM recovery in the range of 3–30 mg/L. The developed test has been used to analyze the concentration of VCM in the blood serum samples (n =108) of patients with extensive burn injuries who were treated with a combined antibiotic therapy. The data obtained were confirmed by the reference LC-MS/MS method, which demonstrated good interassay agreement and did not show the tendency for falsely elevated results observed in a number of immunoassay systems. The data obtained from critically ill patients with burns in our study demonstrate a high risk of suboptimal exposure with standard dosing and potential implications for TDM. Thus, the analytical tool originally developed for the determination of ERM was adapted for the measurement of structurally related VCM. The approach used makes the assay suitable for appropriate therapeutic monitoring of VCM. There are several important future steps required for further development of the assay and its clinical application, which would include dedicated pharmacokinetic studies both in critically ill patients and the general population, assessing the assay performance on other related antibiotics, as well as a full validation procedure.

## Figures and Tables

**Table 1:** Analytical characteristics of ELISA variants based on different coating antigens.

Coating Antigen	IC_50_, ng/mL	IC_20_-IC_80_, ng/mL	IC_10_, ng/mL
BSA-VCM×50f	34.4	6.36–239	1.7
BSA-VCM×50ae	46.1	10.4–271	4.4
Gel-VCM×25pi	22.1	5.0–121	2.1

**Table 2:** Vancomycin recovery from human serum using ELISAs based on different heterologous coating antigens.

Sample Pretreatment	Spiked Level, ng/mL	BSA-VCM×50f		BSA-VCM×50ae		Gel-VCM×25pi	
		RC, %	RSD, %	RC, %	RSD, %	RC, %	RSD, %
PBST	3000	100.3	9.7	96.1	9.3	90.8	7.7
	10,000	89.1	9.8	104.7	12.0	116.5	4.0
	30,000	92.6	11.7	98.1	9.5	114.6	14.2
MeOH	3000	30.1	7.1	15.1	8.1	46.4	8.9
	10,000	50.6	3.0	45.9	11.3	59.9	3.4
	30,000	51.7	8.3	47.9	1.2	58.8	6.1
ACN	3000	96.4	9.7	50.9	7.6	72.3	4.2
	10,000	77.8	6.2	49.1	4.2	73.7	0.7
	30,000	75.0	6.7	72.1	6.2	86.8	2.8
TCA	3000	27.6	10.8	4.2	4.9	35.4	4.8
	10,000	53.5	21.3	6.2	12.0	61.3	4.3
	30,000	63.0	4.0	73.7	4.9	60.1	8.1

RC—mean recovery (n = 4); RSD—relative standard deviation.

**Table 3:** Cross-reactivity (CR) of antibiotics in eremomycin antibody-based ELISA for vancomycin.

Analyte	IC_50_, ng/mL	CR, %
Vancomycin	34.4	100
Eremomycin	33.0	104
Teicoplanin	>10,000	<0.3
Polymyxin B	>10,000	<0.3
Colistin	>10,000	<0.3
Azithromycin	>10,000	<0.3
Amikacin	>10,000	<0.3
Gentamicin	>10,000	<0.3
Tigecycline	>10,000	<0.3
Meropenem	>10,000	<0.3
Linezolid	>10,000	<0.3

## Data Availability

The datasets used and/or analyzed during the current study are available from the corresponding author upon reasonable request.

## Supplementary Materials

The following supporting information can be downloaded at: https://www.mdpi.com/article/10.3390/antibiotics13121133/s1, HPLC-MS/MS procedure, Table S1. HPLC-MS/MS parameters. Figure S1. Chromatograms of VCM (LLOQ 500 ng/mL) (**A**) and IS Doxylamine-d5 (**B**). Sample pretreatment procedure. Table S2. Calibration of spiked human serum samples using HPLC-MS/MS (n = 4). Figure S2. Calibration of spiked human serum samples using HPLC-MS/MS. Table S3. Recovery from spiked human serum samples using HPLC-MS/MS (n = 6). Table S4. Patient clinical and demographic data (n = 4). Figure S3. VCM concentrations in major burn patients (n = 4) obtained using the developed ELISA.

## References

[1] Liu C., Bayer A., Cosgrove S.E., Daum R.S., Fridkin S.K., Gorwitz R.J., Kaplan S.L., Karchmer A.W., Levine D.P., Murray B.E. (2011). Clinical practice guidelines by the Infectious Diseases Society of America for the treatment of methicillin-resistant Staphylococcus aureus infections in adults and children. Clin. Infect. Dis..

[2] Brown N.M., Goodman A.L., Horner C., Jenkins A., Brown E.M. (2021). Treatment of methicillin-resistant Staphylococcus aureus (MRSA): Updated guidelines from the UK. JAC-Antimicrob. Resist..

[3] Belotserkovskiy B.Z., Protsenko D.N., Gelfand E.B. (2018). Antimicrobial therapy of nosocomial pneumonia in era of growth of resistance to carbapenems. Anesteziol. I Reanimatol..

[4] Neely M.N., Kato L., Youn G., Kraler L., Bayard D., van Guilder M., Schumitzky A., Yamada W., Jones B., Minejima E. (2018). Prospective trial on the use of trough concentration versus area under the curve to determine therapeutic vancomycin dosing. Antimicrob. Agents Chemother..

[5] Hale C.M., Seabury R.W., Steele J.M., Darko W., Miller C.D. (2017). Are vancomycin trough concentrations of 15 to 20 mg/L associated with increased attainment of an AUC/MIC ≥ 400 in patients with presumed MRSA infection?. J. Pharm. Pract..

[6] Alobaid A.S., Hites M., Lipman J., Taccone F.S., Roberts J.A. (2016). Effect of obesity on the pharmacokinetics of antimicrobials in critically ill patients: A structured review. Int. J. Antimicrob. Agents.

[7] Dailly E., Le Floch R., Deslandes G., Pannier M., Jolliet P. (2008). Influence of glomerular filtration rate on the clearance of vancomycin administered by continuous infusion in burn patients. Int. J. Antimicrob. Agents.

[8] Del Mar Fernandez de Gatta M., Fruns I., Hernandez J., Caballero D., San Miguel J., Martínez Lanao J. (1993). Vancomycin pharmacokinetics and dosage requirements in hematologic malignancies. Clin. Pharm..

[9] Ocampos-Martinez E., Penaccini L., Scolletta S., Abdelhadii A., Devigili A., Cianferoni S., De Backer D., Jacobs F., Cotton F., Vincent J.-L. (2012). Determinants of early inadequate vancomycin concentrations during continuous infusion in septic patients. Int. J. Antimicrob. Agents.

[10] Colin P.J., Allegaert K., Thomson A.H., Touw D.J., Dolton M., de Hoog M., Roberts J.A., Adane E.D., Yamamoto M., Santos-Buelga D. (2019). Vancomycin pharmacokinetics throughout life: Results from a pooled population analysis and evaluation of current dosing recommendations. Clin. Pharmacokinet..

[11] Kovacevic T., Miljkovic B., Mikov M., Stojisavljevic Satara S., Dragic S., Momcicevic D., Kovacevic P. (2019). The effect of hypoalbuminemia on the therapeutic concentration and dosage of vancomycin in critically ill septic patients in low-resource countries. Dose-Response.

[12] Abdul-Aziz M.H., Alffenaar J.-W.C., Bassetti M., Bracht H., Dimopoulos G., Marriott D., Neely M.N., Paiva J.-A., Pea F., Sjovall F. (2020). Antimicrobial therapeutic drug monitoring in critically ill adult patients: A Position Paper#. Intensive Care Med..

[13] Walker C.A., Kopp B. (1978). Sensitive bioassay for vancomycin. Antimicrob. Agents Chemother..

[14] Do Nascimento P.A., Kogawa A.C., Salgado H.R.N. (2020). Current status of vancomycin analytical methods. J. AOAC Int..

[15] Crossley K., Rotschafer J., Chern M., Mead K., Zaske D. (1980). Comparison of a radioimmunoassay and a microbiological assay for measurement of serum vancomycin concentrations. Antimicrob. Agents Chemother..

[16] Schwenzer K.S., Wang C.-H.J., Anhalt J.P. (1983). Automated fluorescence polarization immunoassay for monitoring vancomycin. Ther. Drug Monit..

[17] Yeo K.-T., Traverse W., Horowitz G. (1989). Clinical performance of the EMIT vancomycin assay. Clin. Chem..

[18] Usman M., Hempel G. (2016). Development and validation of an HPLC method for the determination of vancomycin in human plasma and its comparison with an immunoassay (PETINIA). Springerplus.

[19] Bian L., Liang J., Zhao H., Ye K., Li Z., Liu T., Peng J., Wu Y., Lin G. (2021). Rapid monitoring of vancomycin concentration in serum using europium (III) chelate nanoparticle-based lateral flow immunoassay. Front. Chem..

[20] Scribel L., Galiotto A., Rodrigues I.D.S., Hahn R., Linden R., Zavascki A.P. (2024). Comparison of vancomycin assays in patients undergoing hemodialysis. Braz. J. Infect. Dis..

[21] Liu Y., Mack J.O., Shojaee M., Shaver A., George A., Clarke W., Patel N., Arroyo-Currás N. (2023). Analytical Validation of Aptamer-Based Serum Vancomycin Monitoring Relative to Automated Immunoassays. ACS Sens..

[22] Singer B., Stevens R.W., Westley B.P., Nicolau D.P. (2020). Falsely elevated vancomycin-concentration values from enzyme immunoassay leading to treatment failure. Am. J. Health-Syst. Pharm..

[23] Elzieny M., Fisher J.A., Sims M.D., Lauter C.B., Carey-Ballough R.A., Sun Q. (2023). Falsely decreased vancomycin caused by rheumatoid factor: A case report. Clin. Chim. Acta.

[24] Tsoi V., Bhayana V., Bombassaro A.M., Tirona R.G., Kittanakom S. (2019). Falsely elevated vancomycin concentrations in a patient not receiving vancomycin. Pharmacother. J. Hum. Pharmacol. Drug Ther..

[25] LeGatt D.F., Blakney G.B., Higgins T.N., Schnabl K.L., Shalapay C.E., Dias V.C., Wesenberg J.C. (2012). The effect of paraproteins and rheumatoid factor on four commercial immunoassays for vancomycin: Implications for laboratorians and other health care professionals. Ther. Drug Monit..

[26] Gunther M., Saxinger L., Gray M., LeGatt D. (2013). Two suspected cases of immunoglobulin-mediated interference causing falsely low vancomycin concentrations with the Beckman PETINIA method. Ann. Pharmacother..

[27] Burkin M.A., Galvidis I.A. (2013). Hapten modification approach for switching immunoassay specificity from selective to generic. J. Immunol. Methods.

[28] Burkin M., Galvidis I. (2016). Simultaneous and differential determination of drugs and metabolites using the same antibody: Difloxacin and sarafloxacin case. Anal. Methods.

[29] Burkin M., Galvidis I. (2012). Simultaneous separate and group determination of tylosin and tilmicosin in foodstuffs using single antibody-based immunoassay. Food Chem..

[30] Rybak M.J., Le J., Lodise T.P., Levine D.P., Bradley J.S., Liu C., Mueller B.A., Pai M.P., Wong-Beringer A., Rotschafer J.C. (2020). Therapeutic monitoring of vancomycin for serious methicillin-resistant Staphylococcus aureus infections: A revised consensus guideline and review by the American Society of Health-System Pharmacists, the Infectious Diseases Society of America, the Pediatric Infectious Diseases Society, and the Society of Infectious Diseases Pharmacists. Am. J. Health-Syst. Pharm..

[31] van Hal S.J., Paterson D.L., Lodise T.P. (2013). Systematic review and meta-analysis of vancomycin-induced nephrotoxicity associated with dosing schedules that maintain troughs between 15 and 20 milligrams per liter. Antimicrob. Agents Chemother..

[32] Kullar R., Davis S.L., Levine D.P., Rybak M.J. (2011). Impact of vancomycin exposure on outcomes in patients with methicillin-resistant Staphylococcus aureus bacteremia: Support for consensus guidelines suggested targets. Clin. Infect. Dis..

[33] Cheng X., Ma J., Su J. (2022). An overview of analytical methodologies for determination of vancomycin in human plasma. Molecules.

[34] Smelter D.F., Trisler M.J., McCreary E.K., Baker M., Copeland K., Dilworth T.J., Rose W.E. (2022). Long-Acting Lipoglycopeptides Can Interfere With Vancomycin Therapeutic Drug Monitoring. J. Clin. Pharmacol..

[35] Somerville A.L., Wright D.H., Rotschafer J.C. (1999). Implications of vancomycin degradation products on therapeutic drug monitoring in patients with end-stage renal disease. Pharmacother. J. Hum. Pharmacol. Drug Ther..

[36] Dolton M., Xu H., Cheong E., Maitz P., Kennedy P., Gottlieb T., Buono E., McLachlan A.J. (2010). Vancomycin pharmacokinetics in patients with severe burn injuries. Burns.

[37] Akers K.S., Cota J.M., Chung K.K., Renz E.M., Mende K., Murray C.K. (2012). Serum vancomycin levels resulting from continuous or intermittent infusion in critically ill burn patients with or without continuous renal replacement therapy. J. Burn Care Res..

[38] Burkin M., Burkin A. (2009). Enzyme immunoassay for the determination of the glycopeptide antibiotic eremomycin. Appl. Biochem. Microbiol..

[39] Hermanson G.T. (2013). Bioconjugate Techniques.

[40] Burkin M.A., Galvidis I.A., Eremin S.A. (2019). Specific and generic immunorecognition of glycopeptide antibiotics promoted by unique and multiple orientations of hapten. Biosensors.

[41] Beloborodov V.B., Gusarov V.G., Dekhnich А.V., Zamyatin M.N., Zubareva N.А., Zyryanov S.K., Kamyshova D.А., Klimko N.N., Kozlov R.S., Polushin Y.S. (2020). Diagnostics and antimicrobial therapy of the infections caused by multiresistant microorganisms. Guidelines of the Association of Anesthesiologists-Intensivists, the Interregional Non-Governmental Organization Alliance of Clinical Chemotherapists and Microbiologists, the Interregional Association for Clinical Microbiology and Antimicrobial Chemotherapy (IACMAC), and NGO Russian Sepsis Forum. Messenger Anesthesiol. Resusc..

[42] Surovoy Y.A., Burkin M.A., Galvidis I.A., Sobolev M.A., Rende O.C., Tsarenko S.V. (2023). Comparative polymyxin B pharmacokinetics in critically ill patients with renal insufficiency and in continuous veno-venous hemodialysis. Eur. J. Clin. Pharmacol..

[43] Dijkstra J., Voerman A., Greijdanus B., Touw D., Alffenaar J. (2016). Immunoassay analysis of kanamycin in serum using the tobramycin kit. Antimicrob. Agents Chemother..

[44] Larson T., Gerding D., Peterson L., Eckfeldt J. (1982). Assay of netilmicin, using enzyme immunoassay for gentamicin. Antimicrob. Agents Chemother..

