# Supplementary material for: Therapeutic Monitoring of Vancomycin Implemented by Eremomycin ELISA
Source: Antibiotics (Basel). 2024 Nov 25;13(12):1133. doi: 10.3390/antibiotics13121133 (PMC11672653; doi:10.3390/antibiotics13121133)
Supplement: Supplementary file 1 [file antibiotics-13-01133-s001.zip › antibiotics-3252496-supplementary.pdf]

Supplementary Information

# Therapeutic Monitoring of Vancomycin Implemented by Eremomycin ELISA

**Inna A. Galvidis [1], Yury A. Surovoy [2], Vitaly R. Sharipov [3], Pavel D. Sobolev [3] and Maksim A. Burkin [1,*]**

[1] I. Mechnikov Research Institute for Vaccines and Sera, Moscow 105064, Russia; galvidis@yandex.ru
[2] University College of London Hospital, London NW1 2BU, UK; ysurovoy@gmail.com
[3] Exacte Labs LLC, Moscow 117246, Russia; vitaly.sharipov@exactelabs.com (V.R.S.); pavel.sobolev@exactelabs.com (P.D.S.)
* Correspondence: burma68@yandex.ru; Tel.: +7-495-9172753

## HPLC-MS/MS procedure

An Agilent Infinity 1290 liquid chromatograph equipped with a binary pump and an autosampler was used. Separation was carried out using an Acquity Waters BEH C18 column (50 × 2.1 mm) with an adsorbent grain size of 1.8 μm in a gradient elution mode. The column oven and the autosampler were maintained at 40 and 5°C, respectively, during operation. The analytes were separated using a mobile phase containing 0.1% formic acid in water (eluent A) and 0.1% formic acid in ACN (eluent B). The gradient program was as follows: 0–1.00 min, increase from 5% to 50% B; 1.00-1.05 min, increase from 50% to 100% B; 1.05–2.00 min, 100% B; 2.00–2.05 min, return to 5% B; and 2.05–3.00 min, 5% B. The mobile phase flow rate was 0.5 mL min− 1. The injection volume was 1 μL. A triple quadrupole mass spectrometer AB Sciex QTRAP 5500 mass spectrometer equipped with a Turbo-V™ ion source (AB Sciex, Framingham, MA, USA) was configured to collect data in the multiple reaction monitoring (MRM) mode.

The mass spectrometer equipped with an electrospray ionization (ESI) source was operated in positive ion mode. Nitrogen was used as a nebulizer as well as a curtain gas. The ion source conditions were set as follows: temperature, 550 °C; ion spray voltage, 2500 V; the nebulizer and heat gas pressure, 60 psi; curtain gas pressure, 25 psi; and the collision gas was set to medium flow. Multiple reaction monitoring (MRM) conditions, collision energy (CE), and Cell Exit Potential (CXP) were first optimized for each analyte by injecting solutions of the standards prepared in the ACN/$H_2O$ + 0.1% FA solution. Characteristic molecular ions were selected as precursor ions, and one product ion was monitored for each compound. For quantification, the most intense MRM transition was monitored. MRM parameters and retention times for vancomycin (VCM) and internal standards are provided in Table S1 and the chromatograms obtained are shown in Figure S1.

**Table S1.** HPLC-MS/MS parameters

| Compound | Ionization mode | m/z of precursor ion | m/z of product ions | Declastering potential (V) | Entrance potential (V) | Collision energy (V) | Cell exit potential (V) | Retention time, min[2] |
|---|---|---|---|---|---|---|---|---|
| Vancomycin | ESI+ | 725.0 | 144.2 | 70 | 10 | 21 | 14 | 0.6 |
| (IS) Doxylamine-$d_5$ | ESI+ | 276.1 | 187.3 | 80 | 10 | 20 | 12 | 0.8 |

(IS) Internal standard

**A**

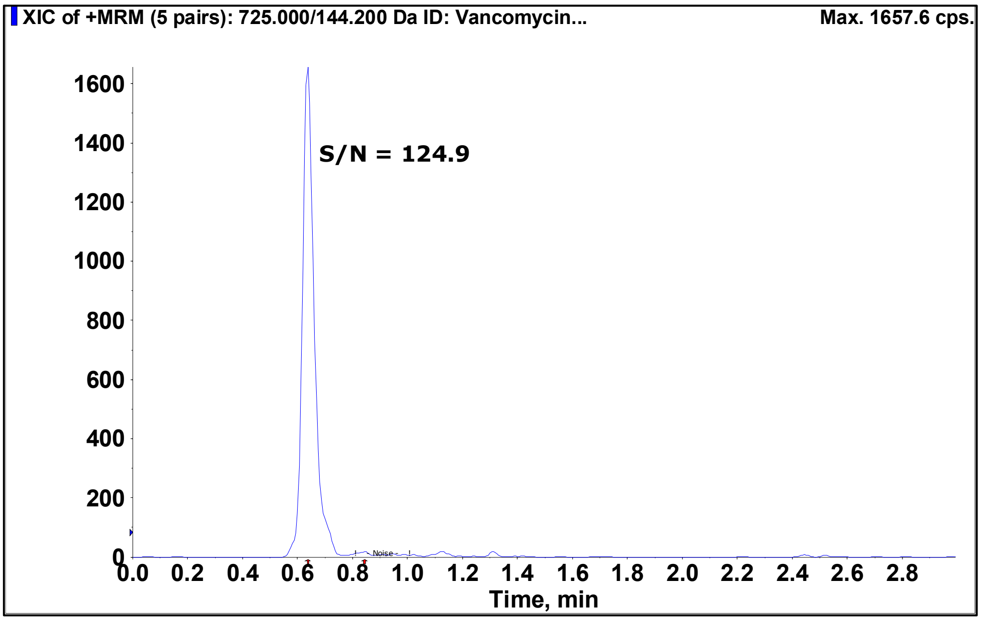


**B**

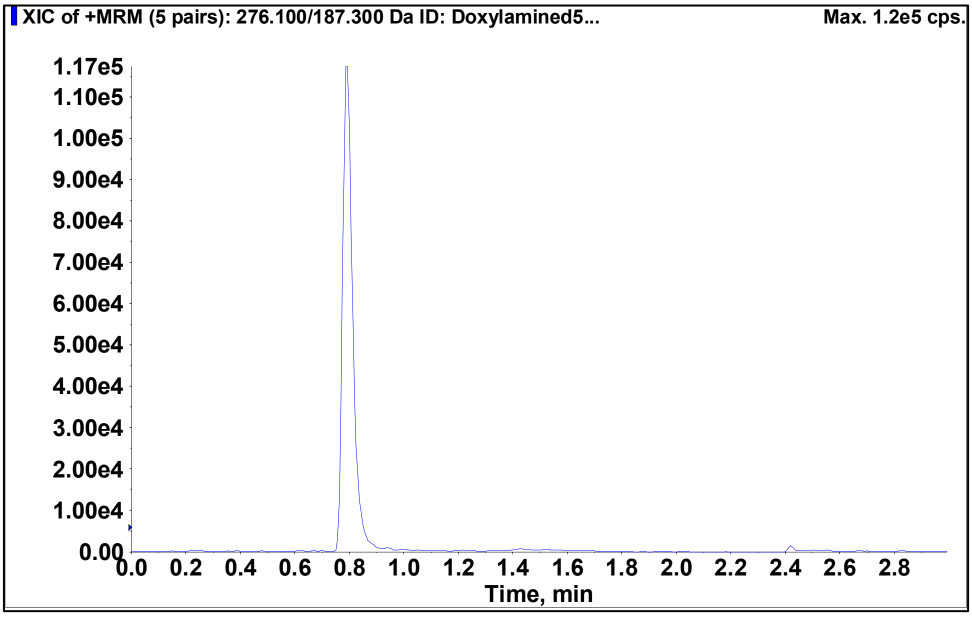


**Figure S1**. Chromatograms of VCM (LLOQ 500 ng/ml) (A) and IS Doxylamine-d5 (B).

## Sample pretreatment procedure

An aliquot (10 µL) of the analyte working solution and an aliquot (10 µL) of the IS working solution (1000 ng/ml) were added to an aliquot (100 µL) of serum. After vortexing the sample, 400 µL of cooled (4 °C) water contacting 15% trifluoroacetic acid was added for precipitation, and the sample was mixed well and centrifuged (2750 × g, 15 min, 4 °C). An aliquot (20 µL) of the supernatant was transferred to a test plate containing 230 µL ACN/$H_2O$ (10/90) solution for further UPLC-MS/MS analysis (Table S2). Calibration was constructed as a function of VCM concentrations versus corresponding peak area values (Figure S2).

**Table S2.** Calibration of spiked human serum samples using HPLC-MS/MS (n=4)

| VCM spiked, ng/mL | Calculated concentration, ng/mL | SD | CV, % | Recovery, % |
|---|---|---|---|---|
| 500 | 513.8 | 63.82 | 12.4 | 103 |
| 1000 | 951.3 | 71.42 | 7.51 | 95.1 |
| 2500 | 2488 | 196.5 | 7.90 | 99.5 |
| 5000 | 4823 | 363.2 | 7.53 | 96.5 |
| 12500 | 12550 | 1226 | 9.77 | 100 |
| 25000 | 25075 | 2123 | 8.47 | 100 |
| 40000 | 40950 | 4455 | 10.9 | 102 |
| 50000 | 51425 | 5749 | 11.2 | 103 |

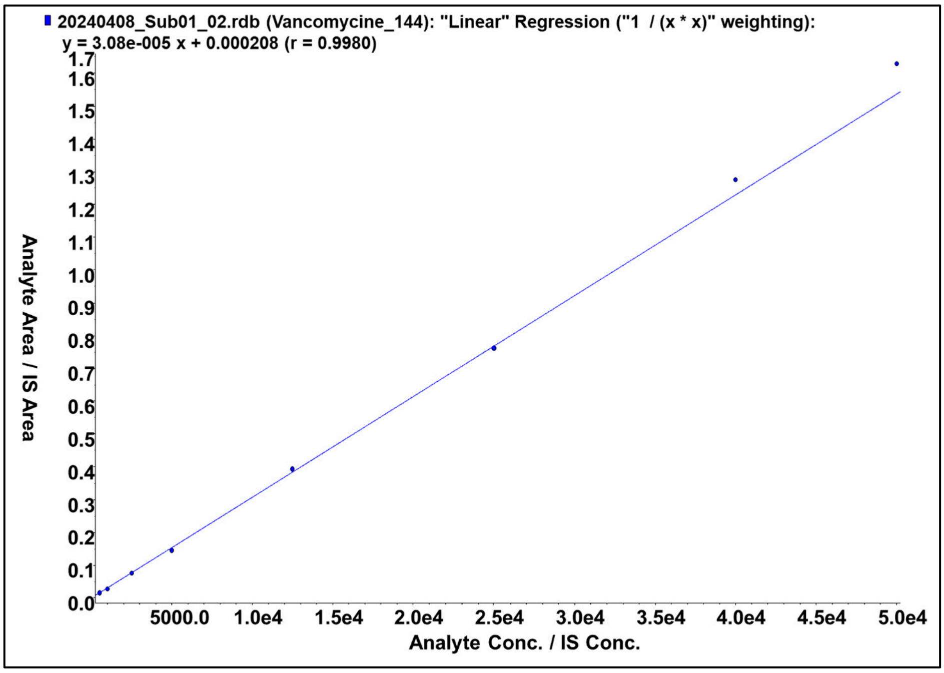


**Figure S2.** Calibration of spiked human serum samples using HPLC-MS/MS

**Table S3.** Recovery from spiked human serum samples using HPLC-MS/MS (N=6)

| Analyte Concentration, ng/mL | | 3000 | 10000 | 30000 |
|---|---|---|---|---|
| Calculated Concentration, ng/ml | Replicate №1 | 3080 | 9110 | 30300 |
| | Replicate №2 | 3070 | 9900 | 30200 |
| | Replicate №3 | 2940 | 9810 | 28300 |
| | Replicate №4 | 3020 | 10300 | 31700 |
| | Replicate №5 | 3090 | 9990 | 30600 |
| | Replicate №6 | 2890 | 10600 | 29800 |
| Mean Concentration, ng/ml | | 3015 | 9952 | 30150 |
| SD | | 82,64 | 504,8 | 1111 |
| CV, % | | 2,74 | 5,07 | 3,69 |
| Mean Accuracy, % | | 101 | 99,5 | 101 |

**Table S4.** Patient clinical and demographic data (n=4)

| Parameter | Patient 1 | Patient 2 | Patient 3 | Patient 4 | Mean |
|---|---|---|---|---|---|
| Age, years | 29 | 46 | 44 | 28 | 37 |
| Weight, kg | 70 | 80 | 75 | 100 | 81 |
| Creatinine, µmol/L | 65 | 80 | 89 | 90 | 81 |
| GFR, mL/min | 147 | 94 | 100 | 153 | 124 |
| Total protein, g/L | 44 | 49 | 64 | 43 | 50 |
| Albumin, g/L | 24 | 28 | 28 | 25 | 26 |
| Isolated bacteria | *E. faecalis* | *E. faecalis* | *S. aureus* | *E. faecalis* | n/a |

GFR – glomerular filtration rate.

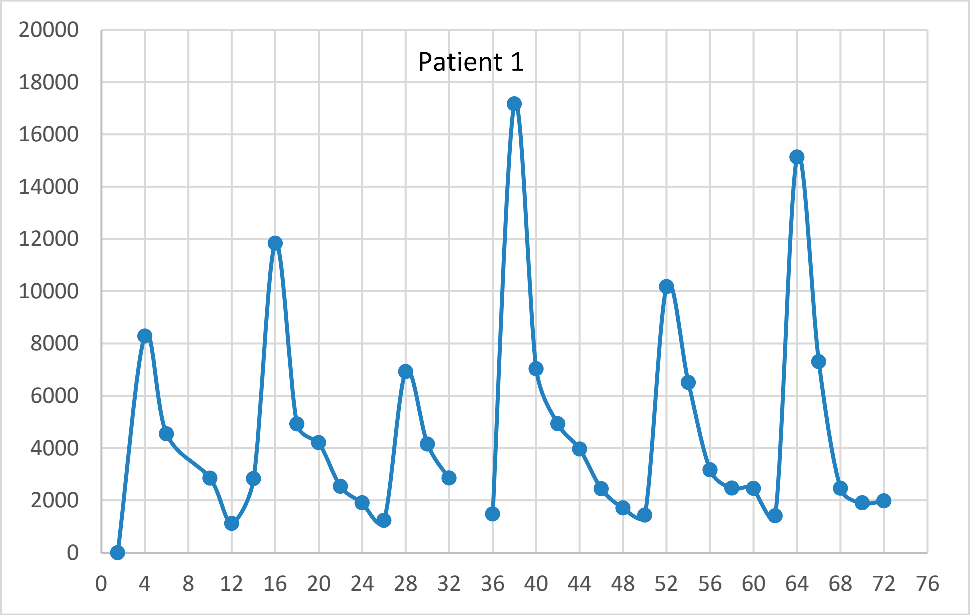

Patient 1
20000
18000
16000
14000
12000
10000
8000
6000
4000
2000
0
0 4 8 12 16 20 24 28 32 36 40 44 48 52 56 60 64 68 72 76


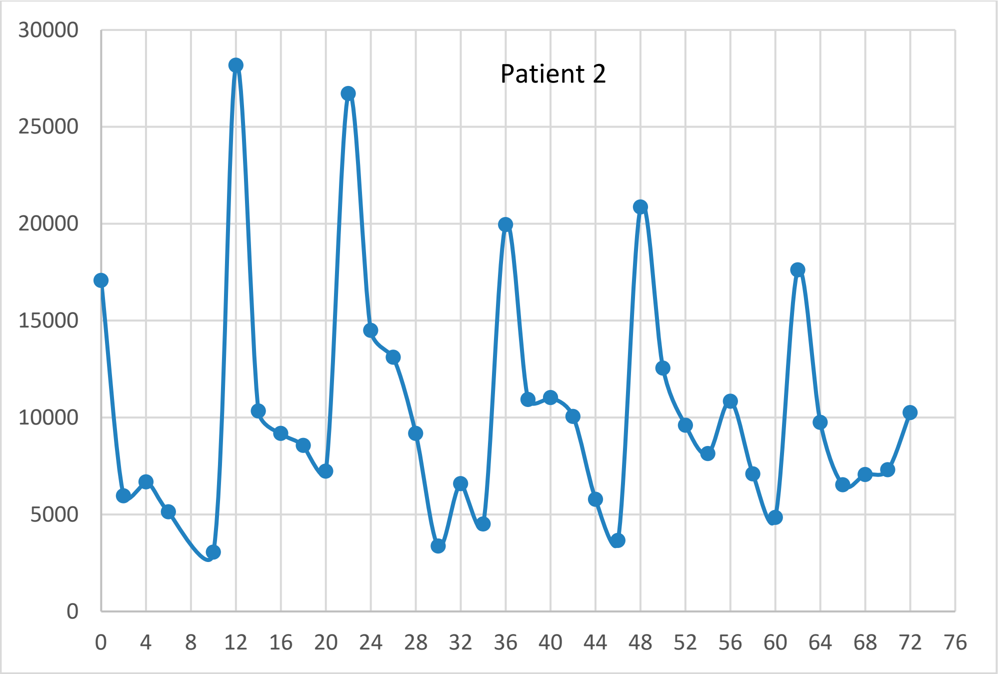

Patient 2
30000
25000
20000
15000
10000
5000
0
0 4 8 12 16 20 24 28 32 36 40 44 48 52 56 60 64 68 72 76

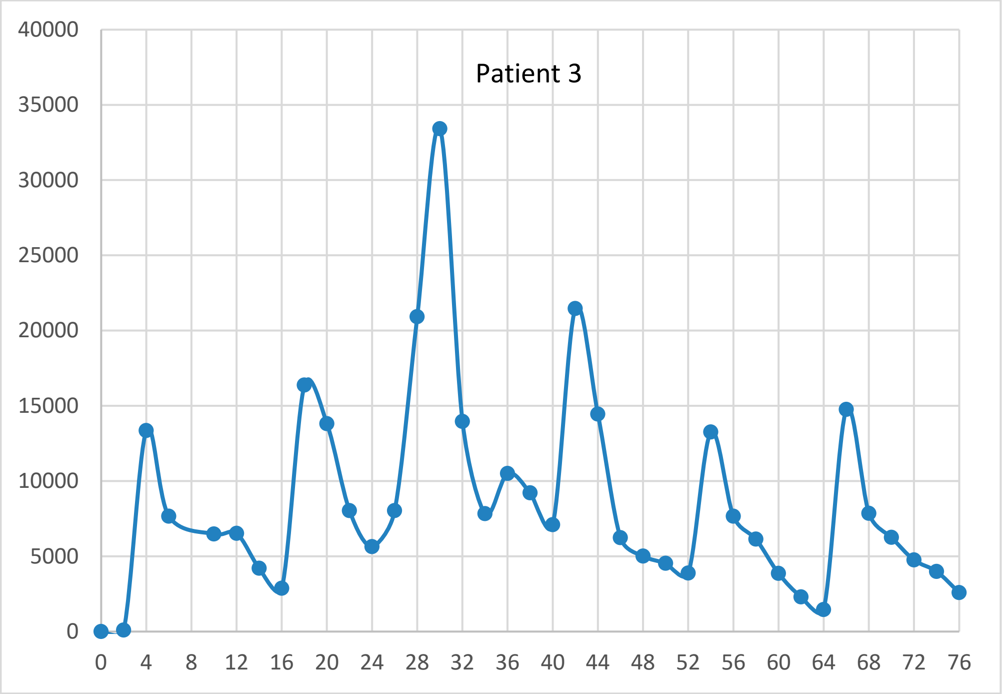


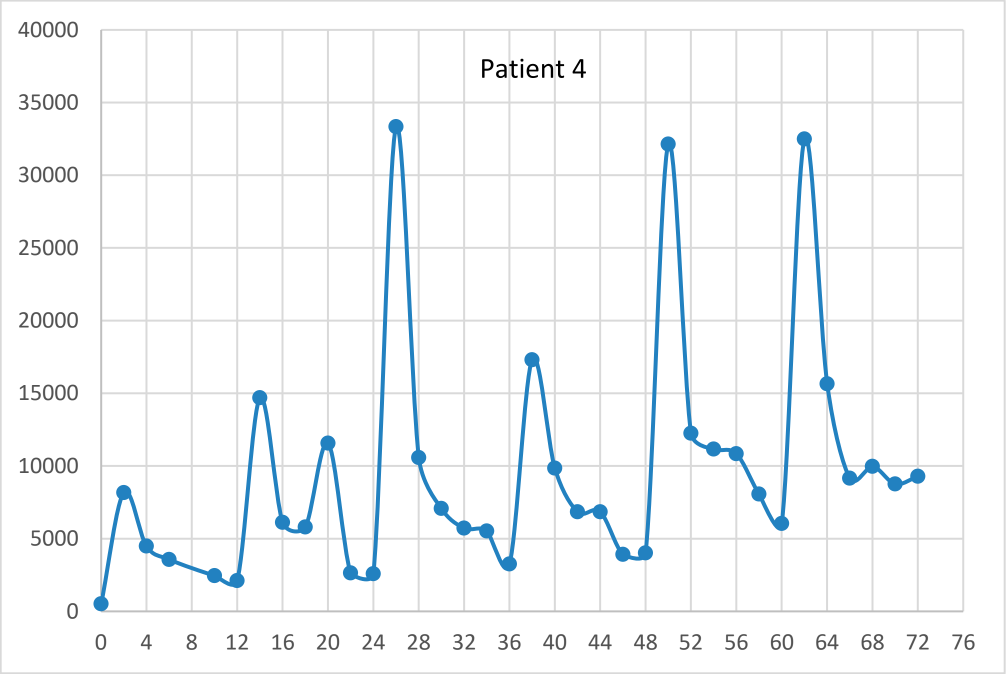


**Figure S3.** VCM concentrations in major burn patients (n=4) obtained using the developed ELISA.
